# Safety in fragile, conflict-affected, and vulnerable settings: An evidence scanning approach for identifying patient safety interventions

**DOI:** 10.7189/jogh.12.04018

**Published:** 2022-02-26

**Authors:** Niki O’Brien, Alexandra Shaw, Kelsey Flott, Sheila Leatherman, Mike Durkin

**Affiliations:** 1 Institute of Global Health Innovation, Imperial College London, London, UK; 2 NIHR Imperial Patient Safety Translational Research Centre, Imperial College London, London, UK; 3 UNC Gillings School of Global Public Health, University of North Carolina, North Carolina, USA

## Abstract

**Background:**

The number of people living in fragile, conflict-affected, and vulnerable (FCV) settings is growing rapidly and attention to achieving universal health coverage must be accompanied by sufficient focus on the safety of care for universal access to be meaningful. Healthcare workers in these settings are working under extreme conditions, often with insufficient contextualized evidence to support decision-making. Recognising the relative paucity of, and methodological issues in gathering evidence from these settings, the evidence scanning described in this paper considered which patient safety interventions might offer the ‘better bet’, eg, the most effective and appropriate intervention in FCV settings.

**Methods:**

An evidence scanning approach was used to examine the literature. The search was limited to FCV settings and low-income settings as defined by the World Bank, but if a systematic review included a mix of evidence from FCV/low income settings, as well as low-middle income settings, it was included. The search was conducted in English and limited to studies published from 2003 onwards, utilising Google Scholar as a publicly accessible database and further review of the grey literature, with specific attention to the outputs of non-governmental organisations. The search and subsequent analysis were completed between April and June 2020.

**Results:**

The majority of studies identified related to strengthening infection prevention and control which was also found to be the ‘better bet’ intervention that could generalise to other settings, be most feasible to implement, and most effective for improving patient care and associated outcomes. Other prioritized interventions include risk management, with contributing elements such as reporting, audits, and death review processes.

**Conclusions:**

Infection prevention and control interventions dominate in the literature for multiple reasons including strength of evidence, acceptability, feasibility, and impact on patient and health worker well-being. However, there is an urgent need to further develop the evidence base, specialist knowledge, and field guidance on a range of other patient safety interventions such as education and training, patient identification, subject specific safety actions, and risk management.

The safety and quality of health care services are increasingly recognised as essential attributes in the provision of universal health coverage (UHC). Patients should not have to make the decision between seeking unsafe care or foregoing necessary medical care. Fragile, conflict-affected and vulnerable settings (FCV) is a term used to describe a range of situations including ‘humanitarian crises, ongoing disruption to public services, significant armed conflict, extreme adversity, or acute, protracted or complex emergencies’ [1]. Health systems in these settings suffer from grave challenges with patient outcomes compromised by inadequate funding and infrastructure, staff shortages and lack of sufficient specialised clinical skills, amidst insecure and unsafe conditions for patients and workers. In addition to these, health systems in these settings often lack adequate governance and health care services may be provided without oversight or regulation of care [2].

Approximately 2 billion people globally live in FCV settings, with nearly 50% of the global poor expected to live in these settings by 2030 due to widespread political instability, conflict, the impact of climate change and most recently, the COVID-19 pandemic which is having a profound impact on health systems and economies around the world [3]. Historically, the focus in these settings has been on providing UHC, however the vulnerability of people in FCV settings makes the quality and safety of the care they receive even more crucial [2]. Reducing avoidable harm is a critical challenge in such settings, where, for example, it is estimated that 60% of preventable maternal deaths globally take place [4]. The World Health Organization (WHO) has recently published several reports with implications for patient safety in FCV settings, including a technical package with tools and resources on quality of care in fragile, conflict- affected and vulnerable settings [4], and the Global Patient Safety Action Plan 2021-2030[5]. To improve the safety and quality of care in these settings, urgent action must also be taken to identify and implement evidence-based interventions. Systematic implementation of these interventions should include those aiming to reduce avoidable harm suffered from health care in these settings.

Those working in FCV settings face formidable challenges to their time and resources, having to make considered, often rapid and pressured, decisions and need to be guided by some form of evidence base to improve the safety of care provided. Provision of a pragmatic evidence base is required to move beyond traditional scientific or academic models due to the challenges in carrying out research in these settings. Further, applying a rigorous, highly academic standard of evidence may not be uniformly possible due to the lack of rigorous research to date [6]. However, and importantly, patients in FCV settings are known to be at increased risk for harm and therefore, the best evidence currently available should be made accessible to inform health care delivery in real time [6].

Strategic objective six of seven identified in the WHO Action Plan is to “Ensure a constant flow of information and knowledge to drive mitigation of risk, a reduction in levels of avoidable harm and improvements in the safety of care” [5]. To provide some progress towards the evidence base and increase information and knowledge, the evidence scanning approach described in this paper provides some initial information as to which interventions may be most effective for improving the safety of care, providing a first step towards building future models for safer care. Notably, evidence scanning should not be considered a standalone or long-term approach to assessing the effectiveness in FCV settings, but rather a pragmatic, interim approach, as global research and health policy stakeholders work towards developing robust and varied methods of evidence production and dissemination in FCV contexts.

The objectives of this study are (1) to review and appraise evidence on an area of patient safety interventions in FCV settings through an evidence scanning approach and; (2) to evaluate the limitations of this approach to inform future refinement.

## METHODS

### Evidence scanning approach

Assessing the quality and safety of health care provided in FCV settings is an emerging field. Research thus far has been relatively unable to use robust methods of evaluation in these complex conditions. However, evidence scanning can provide initial insights into those interventions that may be most useful for improving patient care and reducing avoidable harm. The patient safety interventions utilised for this scanning process were developed through a process of literature review, expert consultation and are explained in the recent WHO publication *Quality of care in fragile, conflict-affected, and vulnerable settings: Taking action *[4]. The report recommends five intervention areas, one of which focused on reducing harm to patients and populations, which, reinforced with other research, and further developed was refined as ‘Reduce avoidable harm’ [7]. This was expanded into four priority areas of intervention:

- Strengthen infection prevention and control (IPC),
- Implement priority patient safety interventions at the point of care,
- Provide hands on patient safety training to health care workers,
- Introduce a context-specific risk management tool.

The evidence scanning approach aimed to provide an initial first step towards considering future models for safer care in FCV settings through developing evidence tables of patient safety interventions for FCV settings using a rapid structured review. The ‘Reduce avoidable harm’ intervention area was selected by the research team as the intervention area to narrow and focus the search to trial the evidence scanning approach and most relevant to their expertise. This particular intervention also had the advantage of being described in detail in the publication (Box 12) [4], making the scope of the search more clearly defined. “Reduce avoidable harm” is further highlighted in the simple gap analysis outlined in *Quality of care in fragile, conflict-affected, and vulnerable settings: Tools and Resources Compendium* as one of two intervention areas where resources exist (in some cases with partial gaps) across all of the sub-intervention areas, which may have facilitated a greater likelihood of research or evidence gathering activities being undertaken to appraise these sub-intervention areas and/or associated tools and resources.

#### Data sources and search

The search and subsequent analysis were completed between May and June 2020. The search included studies reported in English. Google Scholar was searched as a publicly available and accessible database, which contains more grey literature that is not held in traditional academic databases. Additional scanning of the grey literature, particularly the outputs of non-governmental organisations (NGOs) such as Médecins Sans Frontières (MSF), was also undertaken by the researchers. Search terms were developed using the 2019 list of low- and lower-middle income countries (LMICs) as defined by the World Bank Country and Lending Groups [8]. The search focused on FCV and low-income settings but if a systematic review, included low-middle income settings (eg,, Bangladesh, Kenya, Honduras, Nigeria, Pakistan, Timor Leste, West Bank/Gaza, etc.), it was considered. An upper- middle-income country such as Iraq was also included if it was an FCV setting. Search terms for the four sub-intervention areas were developed by the researchers and agreed prior to the search being undertaken by two researchers (AS and NO).

#### Study selection

Studies published from 2003 onwards were reviewed. The search aimed to find the most robust articles in the field, defined as those in a Cochrane review, in peer-reviewed journals with impact, outcome or output data and significance of findings reported. However, the results included were not limited to this. Given the lack of depth and breadth of evidence on the topic of patient safety interventions, specifically interventions to reduce avoidable harm, in FCVs, it was necessary to consider studies which attempt to assess the evidence for an intervention even where this may not be as robust as wished. As such, we included interventions where a minimal number of studies were identified, studies which suggested that an intervention may have potential, and those in which the evidence was less robust.

Two researchers independently searched the selected databases and grey literature across the sub-interventions. The titles and abstracts of articles obtained from the searches were reviewed for relevance to the topic. Papers and reports that did not focus on any of the four sub-intervention areas, or did not meet the country or setting criteria described previously, were excluded. The researchers met to review the full text of selected documents to determine the categorisation of the evidence, where there was a lack of clarity on which sub-intervention the evidence was most applicable to (eg, whether a paper focused on a handwashing intervention should be listed as a point of care intervention or an infection or prevention control intervention).

### Adopting a “better bets” approach to evidence scanning

The intention of the second phase of the evidence review was to both define the interventions with an evidence-base alongside determining what we assessed to be ‘better bet’ interventions for FCV settings. What do we mean by a “better bets” approach? As academics, we understand and appreciate the need for rigorous and evaluative evidence reviews. However, we must also recognize that life and death decisions are constantly being made by policymakers, managers, and frontline providers in FCV settings, often handicapped by inaccessible and incoherent evidence even where directional evidence exists; inadequate resources, infrastructure, and skills to operationalize. Therefore, the existing evidence base needs to be assessed to identify which might be “better bet” interventions to be implemented in austere and adverse health care settings. In this content a “better bets approach” describes the formal and systematic assessment of the existing evidence base to identify interventions most useful and feasible to implement in FCV settings. We acknowledge that the “better bets” approach is a novel method to assess which interventions may be most effective in implementation, summarising the associated biases in the limitations section, but intend to develop the approach and scoring system through further research.

Following completion of the evidence scans, a better bets scoring system was conducted, utilising four criteria as outlined in **Table 1****.** “Generalizability of the studies’ evidence” refers to the extent to which findings can be extrapolated to similar settings[9]. “Feasibility of implementation” addresses whether the interventions are practical and appropriate in the existing or another setting, which is important to establish prior to embarking on implementing a relatively large-scale intervention. “Positive results” as a criterion is important as the literature scan was conducted to find evidence where patient safety interventions were shown to be effective and/or beneficial in some way, especially for FCV settings. Including “positive results” rather than “effectiveness” as a criterion was based on logical rationale, however the limitation is that the rapid evidence scan did not search for the complete universe of articles on the subject, which would encompass a better balance of evidence on what works and what does not work. “Representativeness” refers to the focus on interventions relevant to FCV contexts and health care settings. A single sub-intervention (eg,, ICP) with evidence only representing one health care setting or country, for example, would score lower than an intervention with positive results across multiple countries and/or health care settings. Studies in multiple settings/countries were considered by the authors to be more representative of resource constrained conditions in FCV settings as the interventions were assessed in a range of relevant environments. Again, the limitation of assessing representativeness from results of the rapid evidence scan is that the studies we selected are not necessarily representative of all studies in FCV and low-income settings and thus may be subject to bias.

**Table 1:** Definitions of the better bets criteria

Criteria	Definition
Generalisability of the studies’ evidence	The possibility of extending the research findings from the settings in the identified studies to the wider population and FCV settings, including countries, health care settings, medical specialties, and staff groups.
Feasibility of implementation	The ease and likelihood that the intervention could be applied, focusing on practicality and evaluation of required resources in assessing the feasibility of implementing each intervention in real-world, lived FCV settings.
Positive results	The effect of interventions specifically on health outcomes. Considered to be a range of recognised change measures in sub-interventions, including numeric change, or in qualitative studies a consideration by staff members of a shift in culture or understanding of importance of patient safety.
Representativeness	Consideration of how reasonably representative the studies were across FCV settings, including facility types, and geographical settings; eg, studies conducted solely in FCV settings, or covered a variety of geographical areas, some or all of which were FCV settings, given higher scores than those conducted in non-FCV settings only, or one geographical area.

FCV – fragile, conflict-affected, and vulnerable

The scoring took place in June 2020. Three reviewers, Niki O’Brien (MSc), Alex Shaw (MSc), and Mike Durkin (FRCA, FRCP, DSc), independently examined each intervention evidence table and applied these four criteria based on a rating scale of 0,1 or 2. Each reviewer independently scored each intervention against these criteria and then scores were totalled to a maximum of 8 points. Comparing the scores, a discrepancy of 2 or more points was considered disagreement. In such cases each reviewer’s rationale for giving the score was presented and each reviewer was able to adjust their score if they considered it necessary based on the rationales presented. The scores were then averaged to produce a mean score. A score of 7 or 8 was anticipated to be a reasonable threshold to identify better bets. The fact that this threshold was not attained cannot be judged as a conclusive assessment of the merit of the interventions but rather may be indicative of the quality and quantity of studies.

### Role of the funding source

This work was supported via the NIHR Imperial Patient Safety Translational Research Centre, Institute of Global Health Innovation, alongside input from the UNC Gillings School of Public Health. No other funding sources were utilised.

## RESULTS

The evidence scanning process resulted in 34 studies being identified across each of the four ‘reduce avoidable harm’ interventions (see full list in Tables S1-S4 in the **Online Supplementary Document**) [10-43]. The studies were predominantly carried out in LMICs rather than FCVs. Seven studies were identified as having taken place in a country categorised within the World Bank Harmonized List of Fragile Settings: Democratic Republic of Congo, Kiribati, Kosovo, Papua New Guinea, Republic of Congo, Solomon Islands and Sudan. There was a noticeable trend in the year of publication with most studies found published after 2016, with a limited number published between 2011-2015 as seen in **Table 2**, marking the recent nature of the growth in this area of research.

**Table 2:** Number of publications by year

Year of publication	Total number of publications (n)
<2010	1
2011-2015	6
2016-2020	27
**Total**	**34**

The most articles were found related to the broad categories of interventions for strengthening infection prevention and control (N = 12), followed by providing hands on patient safety training to health care workers (N = 9) and introduction to a context-specific risk management tool (N = 7). Notably, the fewest number of studies were found for the implementation of interventions to reduce avoidable harm at the point of care (N = 6). There were some challenges in the categorisation of interventions related to training. While the majority of interventions related to training were found in the search for hands on patient safety training for health care workers (and subsequently recorded as interventions in this category), interventions that partly or wholly relied on training were also found and reported in searches for the other priority areas. Of these, five studies reported training interventions in the strengthening ICP priority area, two studies reported training as interventions categorised under point of care and one study utilised training as a risk-management tool intervention. The results were organised and described to outline the sub-interventions, methodology and the results and outputs. The interventions tables developed from the evidence scanning are shown in Tables S1-S4 in the **Online Supplementary Document**.

### Better bets results

After the better bets scoring was totalled, none of the interventions evaluated for ‘reducing avoidable harm’ yielded an average score of 7 or 8 as seen in **Table 3**. Although none of the interventions reached the threshold of inclusion in the better bets list, infection prevention control was the intervention that was considered to hold the most promise as a better bet for patient safety intervention for reducing avoidable harm in FCV settings. The conclusion from this process was that more research was needed to know which interventions would be most effective in this environment.

**Table 3:** Results from scoring the interventions against the better bets criteria

Intervention	Scorer 1	Scorer 2	Scorer 3	Average score
Infection prevention control	7	7	5	6.33
Priority Interventions at the Point of Care	3	2	4	3
Patient Safety Training for Healthcare workers	5	5	6	5.33
Risk management	7	5	5	5.66

## DISCUSSION

We found relatively few studies on patient safety interventions for reducing avoidable harm in FCVs, particularly before 2016, suggesting that this area of patient safety research is a relatively new field of study. The studies identified varied in their area of focus, methodology and geographic focus, which again indicates that research on patient safety interventions for reducing avoidable harm in FCV settings is in its infancy. However, the authors note that given the topic area, it is possible that more work to evaluate safety interventions has been done at the national or facility level in-country, but the results have not been published, or not published widely enough to be picked up internationally. It is only possible to understand the full extent of avoidable harm interventions, and patient safety interventions and their effectiveness in FCVs more widely, by engaging with in-country stakeholders, including hospital and medical centre administrators and clinicians. Future research and engagement must align with the eight elements of national quality policy and strategy outlined in *Quality of care in fragile, conflict-affected, and vulnerable settings: Taking action* [4], as well as the WHO Global Patient Safety Action Plan [5], but adapt to individual contexts given the variability between and within FCV settings [6].

### Focus areas in patient safety

Despite the relatively small number of results there is a clear focus on infection prevention and control in the published literature, which accounted for 12 of the 34 publications identified. Such a focus may reflect the global discourse and promotion of infectious disease prevention through initiatives such as the “My five moments for hand hygiene” campaign developed and championed by WHO [44]. Notably, infection and prevention control also received the highest better bets scoring (**Table 3**). This is perhaps not surprising given the better bets scorers require evidence to provide a score in areas such as feasibility of implementation and generalisability of the studies evidence. As such, when particular focus areas have a wealth of evidence available, scoring, particularly lower scores, better reflect the effectiveness of the intervention itself rather than the lack of evidence.

Priority interventions at the point of care was the focus area with the least number of identified studies on effectiveness (6/34) as well as the intervention area with the lowest better bets scoring. Again, this suggests a relationship between the number of studies found and the feasibility of better bets scoring offering a true score of effectiveness. However, the lack of studies found in this topic may also suggest that point of care interventions are not a focus of patient safety interventions to reduce avoidable harm in FCV settings. There are several reasons this could be the case. The most obvious hypothesis is that global patient safety campaigns typically focus on single actions or preventative outcomes such as the second global patient safety challenge ‘Safe Surgery Saves Lives’ [45]. ICP recommendations and guidance, for example, have been promoted widely at the national and international level since the 1980s [46]. Instead, patient safety at the point of care covers multiple actions and preventive outcomes, and is highly context specific. As such, the majority of patient safety interventions at the point of care may not be considered a priority in FCVs as they require greater planning and coordination and are currently without easily accessed resources from health bodies such as WHO.

### Growing attention on FCV settings

A significant number of the papers found through the evidence scanning process were published between 2016 and 2020 (N = 27), with a noticeable proportion also published between 2011-2015, compared to the number found between 2003, as intended by the search parameters, and 2010. There has been a significant change in the prevalence of violent conflict during this period, which has been particularly dramatic since 2010. For example, the onset of the Arab Spring in 2010, followed by the longstanding Syrian and Yemeni civil wars beginning in 2012 and 2014 respectively, has shifted the geographical focus of violent conflict to the Middle East [47]. In Africa, armed conflict has been in decline since 2015, however violence involving militant Islamic groups remains a source of conflict across the continent [48]. Simultaneously, the rise of social media platforms such as Facebook being exclusively accessed on mobile phones increased in this period (since 2012); perhaps offering individuals living and working in FCVs the opportunity to broadcast their experiences more widely for the first time, and bringing attention to the need for research in health and humanitarian research [49].

The global FCV landscape has rapidly changed since this time, due to climate change, increasing inequalities, demographic changes, and political instability [3]. LMICs, where there is likely to be a higher incidence of FCV settings, disproportionately bear the burden of this increasing conflict. The political instability present in the Middle East over the last decade, and in particular the Syrian conflict, alongside a significant increase in the number of displaced people, estimated to be 79.5 million at the end of 2019 [50], could be the cause of this more recent focus on implementing safety interventions for the health care received in LMICs and FCV settings [51]. As such, there is much to learn from effective patient safety interventions in a range of health care settings, including those in LMIC countries. As in FCV settings, health care resources in LMIC settings are often challenged by inadequate resources, infrastructure, and skills to operationalize to varying extents, which could provide a relevant evidence base that could be considered and applied in FCV contexts. However, future research should also seek to advance the evidence base by exploring what interventions may be effective, as well as the facilitators and barriers to successful implementation, across different areas of health care and disaster response research in FCV settings which are likely to have unique challenges.

The COVID-19 pandemic is going to increase the number of extreme poor globally and is exacerbating existing FCV settings. Until now the full impact of the pandemic on health care provision in FCV settings is yet to be quantified but it can be considered, given that it will be these populations who will be disproportionately impacted due to a lack of resources, that these settings are likely to see a long-term impact on health outcomes and provision of safe care as a result.

### Limitations of the evidence scanning and better bets methodology

We recognise the considerable limitations around the evidence scanning and better bets approach and view this work as a starting point to answer a complex problem for those working in these extremely challenging settings. The importance of further research on patient safety interventions in FCV settings and how best to dissect existing evidence given the varied range of challenges and health systems found within and across FCV settings cannot be stressed enough. The primary challenge to the effectiveness of the approach was the lack of existing literature relevant to FCV settings on the selected patient safety topic area: reducing avoidable harm. This is likely due to both a historical lack of funding and research and the challenges of conducting impact research in these settings, but also suggests that much of the relevant research is not being captured in academic publications or accessible grey literature [6]. It was considered whether there is research done in these localities that does not reach peer-reviewed journals or widely published literature that could be accessed and utilised in this area. The limited findings of the evidence scanning process, led us to consider whether it would be beneficial to utilise an expert consultation process where experts working in FCVs have the opportunity to share their view on the effectiveness of specific patient safety interventions for these settings. We believe that health workers and experts working in the field are best placed to advise on where evidence of the effectiveness of patient safety interventions in FCVs can be found, how the effectiveness of interventions can be measured, and where the essential gaps in research lie.

The evidence scanning method was also broad, which as a positive, offered researchers more flexibility in how and where to find evidence, however further definition of keywords and search databases may be beneficial. Another limitation found was the broad nature of the four ‘reducing avoidable harm’ interventions identified by WHO and the lack of a clear distinction between the interventions in some cases (eg, where handwashing could be considered a point of care intervention and an intervention to strengthen ICP. A training course on strengthening ICP could also be categorised as providing hands on training to health care workers). It would be valuable to further vet and refine these interventions, informed by ongoing research and other engagement with those in FCV settings. The evidence scanning also found a wide variety of sub-interventions within each category; it would be beneficial to expand the number of interventions to search within smaller categories to better capture the specificities and scope of patient safety interventions and their effectiveness in future research. As also noted, the approach based on the WHO ‘reducing avoidable harm’ interventions resulted in the inclusion of training interventions across all categories, not just in the hands-on patient safety training for health care workers priority area. It may be helpful to develop hands on patient safety training for health care workers as a sub-category under the other priority areas to reflect that training will be an integral component of all patient safety priorities rather than a standalone priority. The limitations of the better bets scoring process centre around two themes; broadness of the interventions and lack of evidence. The broad nature of the interventions makes it challenging to score them both in comparison with each other and against the four criteria. For future work, and where a larger number of studies might be available, it may be beneficial to group the studies found by the evidence scanning into sub-interventions before undertaking the scoring process.

### Limitations of the paper

There are several limitations that must be acknowledged. While the evidence scanning approach was used to enable a wide and flexible search, relevant papers may still have been missed and evidence selection bias could have occurred during the evidence scanning process. Additionally, in using only one of five areas of interventions as defined by the WHO *Quality of care in fragile, conflict-affected, and vulnerable settings: Taking action report* [4], the results of the evidence scanning may not be reflective of the amount of evidence available in other intervention areas. Finally, there is a risk of selection bias as the study was conducted by researchers in the UK and US, some of whom have limited practical experience delivering health care or patient safety interventions in FCV settings. This limitation was partly mitigated based on the diverse academic background and perspective of the authorship and by following a clearly defined process of better bets scoring. However, future research in this area should seek broader diversity within the research team to ensure clinical and non-clinical health care staff, policymakers, and researchers working in FCV settings are able to contribute to research design and execution.

## CONCLUSIONS

With increasing numbers of the global poor expected to live in FCV settings by 2030 due to widespread political instability, conflict, impacts climate change and the effects of the COVID-19 pandemic, further research on patient safety in such settings is urgently and rapidly needed. The WHO estimates 60% of preventable maternal deaths globally take place in FCV settings, highlighting the need to address avoidable harm and scale up patient safety interventions [4]. Scanning existing evidence on patient safety interventions in FCV settings has facilitated some limited insights into which interventions may be most feasible and effective to implement in FCV settings.

Our findings, unsurprisingly, were that infection prevention and control was the most reported patient safety intervention in the studies relevant to FCV settings. There is an implication from our findings that system and population level interventions can tend to take precedence in FCV settings over individual interventions such as patient identification, possibly due to the increased complexities with implementing micro interventions. However, clearly there is a compelling need to expand the types of safety interventions routinely implemented. Examining the evidence alongside and through the experience of those working in health systems in FCV settings will allow for an expanded understanding of which set of patient safety interventions should be prioritized for provision of both UHC and safe, quality care for populations in FCV settings. There is also a need to examine how existing evidence on interventions in FCVs relate to organisational and system level patient safety objectives, and with the seven strategic objectives outlined in the WHO Patient Safety Action Plan, as well as the WHO Framework for Quality in Fragile States.

As noted, the clear next step is to engage with those working in FCV settings, particularly those in the clinical environment, to understand whether information on the effectiveness of patient safety interventions might exist. Following that, stakeholders such as patients and communities/ clinicians, policy makers and NGOs must also be engaged to assist the global health community more widely in understanding the priority areas for future research in patient safety interventions in FCV settings. However, perhaps the most essential element of future work, and a key consideration throughout the process of identifying next steps, is to ensure research and evidence is developed in such a way that it is free, easily accessible and implementable for those working in already under resourced, fragmented, and overstretched health care environments.

## Additional material


Online Supplementary Document
