# Supplementary material for: Safety in fragile, conflict-affected, and vulnerable settings: An evidence scanning approach for identifying patient safety interventions
Source: J Glob Health. 2022 Feb 26;12:04018. doi: 10.7189/jogh.12.04018 (PMC8876158; doi:10.7189/jogh.12.04018)
Supplement: Online Supplementary Document [file jogh-12-04018-s001.pdf]

Supplementary Document

Table S1. Intervention: Strengthen infection and prevention control

	Method	Description	Evidence Summary	Source	Location and Study Period
1	Infection and prevention control programme (IPCP) implementation	Adoption of IPCP evaluation tool (based on WHO core components) into the Kiribati health environment	Pilot survey using new IPCP Evaluation tool conducted along with survey of HCWs' knowledge of, application of, and confidence in infection control principles.<br>Findings:<br>- Levels of compliance in each area were:<br>- Organisation (100%)<br>- Epidemiologic surveillance (35.3%)<br>- Microbiology (83.3%)<br>- Intervention strategies (76.3%)<br>- Sterilisation and high level disinfection (87.5%)<br>- Personnel health (78.6%)<br>- Hospital environment and sanitation (60.9%)<br>- Healthcare worker survey findings<br>- The mean knowledge score on ICP was 9.23 ± 1.896 out of 15<br>- The mean knowledge score on infection control precautions was 7.54 ± 2.194 out of 12<br>- The mean confidence in infection control precautions was 6.34 ±1.956 out of 8	Zimmerman PA, Yeatman H, Jones M. Murdoch H. Evaluating infection control: A review of implementation of an infection prevention and control program in a low-income country setting. Am J Infect Control. 2013; 41:317-321.	Kiribati<br><br>2010

2	Training in IPC and provision of equipment	Refresher training of healthcare workers in IPC, ensuring adequate supplies and availability of IPC kits (PPEs & IPC supplies) and IPC mentoring of healthcare workers.	Research assessed the impact of HCWs capacity building in basic infection prevention and control (IPC), IPC kit donation and IPC mentoring. The analytical study was designed to allow for before and after comparisons. Data was collected by IPC specialists using an IPC scorecard developed by WHO office for Africa. 48 Healthcare facilities were evaluated and 878 healthcare workers trained.<br>Findings:<br>- Mean IPC score at baseline: hospitals (8%), medical centres (4%), health centres (4%).<br>- Mean IPC score at follow up increased: hospitals (50%), medical centres (39%), health centres (36%) ($p < 0.001$).<br>The aggregate mean score at baseline for all health facilities was 4.41% and at follow-up it was 39.51% (95% CI: 30.85% to 39.31%, p value<0.001).	Ousman K, Kabego L, Talisuna A, Diaz J, Mbuyi J, Houndjo B, et al. The impact of Infection Prevention and control (IPC) bundle implementation on IPC compliance during the Ebola virus outbreak in Mbandaka/ Democratic Republic of the Congo: a before and after design. BMJ Open. 2019;9(9):e029717.	Equateur province, Democratic Republic of the Congo (DRC)<br><br>June-July 2018
3	Checklist based quality improvement programme	Introduction of Clean Cut, a checklist-based quality improvement programme to improve compliance with best practice in preventing surgical site infections (SSI) at tertiary hospital in Ethiopia	Process mapping of infection prevention activities was used to identify a strategy for improving surgical safety. Processes were mapped by visiting surgical fellow and local operating theatre staff to facilitate development of contextually relevant solutions.<br>Findings:<br>- Process mapping helped identify barriers to compliance with a variety of interventions and identify opportunities for improving adherence and developing solutions resulting in high compliance with antiseptic standards.<br>- Compliance with most processes improved significantly following organisational changes. E.g.<br>- Hand and surgical site decontamination increased (24.1% vs 67.6%, p<0.0001)	Forrester JA, Koritsanasky LA, Amenu D, Haynes AB, Berry WR, Alemu S, et al. Developing Process Maps as a Tool for a Surgical Infection Prevention Quality Improvement Initiative in Resource-Constrained Settings. J Am Coll	Ethiopia<br><br>August 2016 – March 2017

			- Compliance of sterile indicator inside instrument tray increased (65% vs 98%, p< 0.0001)<br>- There was significant improvement in timing of antibiotic administration (12.4% vs 23.9%. p= 0.003038)<br>- There was significant improvement in counting swabs before incision (51.5% vs 90.1%)<br>- There was improvement in announcing planned operation (63.%% vs 79.5%, p=0.00377)	Surg. 2018;226(6): 1103-1116.	
4	Training on hand hygiene	Training on the WHO Hand Hygiene multi modal campaign was undertaken across Kosovo	A one-week training course was held for 691 healthcare workers across Kosovo. Training to healthcare workers was provided in the form of lectures, videos and hands-on practical lessons (e.g. cleaning hands with antimicrobial soap). Questionnaires distributed to healthcare workers before and after one week of training. Measurement realised through a 5 point Likert scale.<br>Findings:<br>- Knowledge of healthcare workers differed significantly before and after the training.<br>- The average value of improvement of HCWs knowledge was 41.66% (p<0.002).<br>- The total Score Index Pre-training 12.65 ± 2.47 and Total Score Index Post-training increased to 17.91 ± 2.59	Sopjani I, Jahn P, Behrens J. Training as an Effective Tool to Increase the Knowledge About Hand Hygiene Actions. An Evaluation Study of Training Effectiveness in Kosovo. Med Arch. 2017;71(1):16-19.	Kosovo<br><br>February 2016

5	IPC training	Intensive five day IPC training with focus on Ebola organised by Ministry of Health (MoH) and partners (WHO/ CDC)	All health facilities in Ratoma municipality evaluated on IPC performance standards developed by MoH. Two questionnaires used corresponding to IPC milestones.<br>Findings:<br>- Cascade training implemented in 73% of health centres and IPC score more likely to be above median when health centre had cascade training ($p < 0.001$)<br>- Overall IPC more likely to be above median in public facilities compared to private ($p < 0.01$)<br>- An IPC score above median positively associated with number of IPC trained staff. Health centres with one trained individual more than four times as likely to have IPC score above median than those with no staff	Keita M, Camara AY, Traore F. Impact of infection prevention and control training on health facilities during the Ebola virus disease outbreak in Guinea. BMC Public Health. 2018;18:547.	Guinea<br><br>February 2016
6	Implementation of clean care delivery kits	The kits included soap and gauze/spirit for clean cord care, a plastic sheet, a clean razor blade and clean string for tying the umbilical cord of neonatal infants post-delivery.	The study focused on healthcare facility water, sanitation and hygiene (WASH) services and practices in LMICs. The authors identified a case control study in Raza and Avan, Pakistan assessing the use of clean care delivery kits by healthcare workers in two tertiary care hospitals.<br>Findings:<br>- Hygiene interventions are consistently protective against healthcare associated infections in LMICs.<br>- In Pakistan, after adjustment for socio-economic factors, clean delivery kits were independently associated with reduced NNT (Adjusted matched OR 2.0: 95% CI 1.3-3.1).<br>- Association with clean delivery kits remained significant when additionally adjusted for attendance of skilled birth attendants (amOR 2.0; 95%CI 1.0-3.9, $p=0.05$).	Watson J, D'Mello-Guyett L, Flynn E, Falconer J, Esteves-Mills J, Prual A, et al. Interventions to improve water supply and quality, sanitation and handwashing facilities in healthcare facilities, and their effect on healthcare-associated infections in low-income and middle-income countries: a systematic review and supplementary scoping	Multiple (Pakistan relevant)<br><br>April 2018

				review. BMJ Glob Health. 2019;4: e001632.	
7	Training programme and application of guidelines	Application of training programme with designing of guidelines for infection control in the operating theatre.	The research was undertaken in Sudan with 126 healthcare workers included. The research team used a structured questionnaire, observation and checklist, surgical site infection surveillance sheet, pre and post training tests and in depth interviews.<br>Findings:<br>- Reduction in infection rates found after training programme intervention.<br>- Rate of all cardiothoracic surgical site infections reduced 16% in the first year and 14% in the second year (p=0.05).<br>- Rate of SSI among clean procedures deceased from 18% to 11%	Basheer Koko Baraka, M, Elsayed A, Abdalla Mohamedani AA. Effect on rates of surgical site infection following application of an infection prevention and control training programme in Elsha'ab hospital, Khartoum, Sudan. Journal of US-China Medical Science. 2014;11:205-211.	Sudan<br><br>2010-2011

8	Review of hand hygiene (HH) strategies	Hand Hygiene strategies implemented through WHO SAVE LIVES program	Review effective strategies implemented during last 10 years in developing countries with focus on strategies that have improved hand washing practices in hospital settings.<br>Findings:<br>Mali: WHO multimodal strategy: also locally produced hand rub used. Compliance with HH guidelines improved 8-21.8% at follow up.<br>Rwanda: WHO multimodal strategy: pharmacy distributed hand sanitiser/ translation of guidelines to local language. Compliance with HH increased 66-76% (p=0.002). Stable water supply through installing movable sinks. HH compliance increased by 35%<br>Sudan: WHO multimodal strategy and use of 2009 change model. HH compliance improved from 55-97% 3 weeks post intervention<br>Many other countries experienced improvement in HH compliance after implementation of WHO multimodal strategy.	Matar MJ, Moghnieh RA, Awad LS, Kanj SS. Effective Strategies for Improving Hand Hygiene in Developing Countries. Curr Treat Options Infect Dis. 2018; 10:310-329.	Multiple<br><br>2018
9	Review of surgical interventi on program me	Surgical intervention programme to reduce surgical site infection (SSIs) in Ethiopia	Clean cut (surgical intervention programme) piloted a SSI intervention programme at a tertiary hospital. The primary outcome measure was SSI, with secondary outcomes including other infection, re-operation and length of stay.<br>Findings:<br>- 50 patients recorded prospectively, rates of SSI 14%, re-operation 6%, death 2%.<br>- Compared with direct observation, chart review had high loss to follow up (28%) and decreased capture of infectious complications with SSI 5%, re-operation 1% and death 1%.<br>- Direct follow up is essential, chart review does not accurately reflect post-operative complications.	Forrester JA, Koritsansky L, Parsons BD, Hailu M, Amenu D, Alemu S, et al. Development of a Surgical Infection Surveillance Program at a Tertiary Hospital in Ethiopia: Lessons Learned from Two Surveillance Strategies. Surg Infect. 2018;19(1).	Ethiopia<br><br>September 2016

10	Rapid Improvement Model (RIM) quality improvement (QI) initiative	A 37-item checklist was developed to monitor environmental cleanliness, waste disposal and personal protective equipment (PPE) performance in Sierra Leone	A Rapid Improvement Model (RIM) quality improvement (QI) initiative with a compressed timeframe of 6 months to improve environmental cleanliness, waste disposal and personal protective equipment (PPE) at eight purposively selected health facilities (HFs) in Sierra Leone. HF teams received QI training and weekly coaching and convened monthly to review progress and exchange best practices. At the final learning session, a "harvest package" of the most effective ideas and tools was developed for use at additional HFs.<br>Findings:<br>- The intervention resulted in marked improvement in waste disposal and environmental cleanliness performance and modest improvement in PPE<br>- Aggregate compliance for the 37 indicators increased from 67 to 96% over the course of 4 months, with all HFs showing improvement<br>- PPE compliance improved from 85 to 89%, waste disposal from 63 to 99%, and environmental cleanliness from 51 to 99%.	Rondinelli I, Dougherty G, Madevu-Matson CA, Toure M, Akinjeji A, Ogongo I, et al. An innovative quality improvement approach for rapid improvement of infection prevention and control at health facilities in Sierra Leone, Int J Qual Health Care. 2020;32(2):85-92.	Sierra Leone<br><br>2017-2018

11	WHO Hand Hygiene Self-Assessment Framework (HHSAF)	The HHSAF is a closed-formatted, self-administered questionnaire structured with 27 indicators that reflect the five components of the WHO Multi-modal Hand Hygiene Improvement Strategy.	The 27 indicators of the HHSAF reflect the five components of the WHO Multi-modal Hand Hygiene Improvement Strategy: system change, training and education, performance evaluation and feedback, reminders in the workplace, and institutional safety climate. HCFs worldwide were invited to participate. They were approached via e-mail using a WHO database of approximately 13,000 HCFs in 2011 and 20,000 in 2015, namely those that had registered to the 'SAVE LIVES: Clean Your Hands' campaign. 2119 health facilities from 69 countries participated, and in 2015, 807 health facilities from 2011 - 2015, 91 countries participated.<br>Findings:<br>- Overall score increased significantly from 335.1 to 374.4.<br>- In terms of WHO regions, the scores for the Eastern Mediterranean, Europe and Western Pacific regions all improved significantly.	Kilpatrick C, Tartari A, Gayet-Ageron J, Storr J, Tomczyk S, Allegranzi B. Global hand hygiene improvement progress: two surveys using the WHO Hand Hygiene Self-Assessment Framework, J Hosp Infect. 2018;100(2): 202-206.	Multiple<br><br>2011 & 2015
12	ICP interventions for nurses	Implementation strategies for infection prevention and control promotion for nurses in sub-Saharan Africa	The purpose of this review is to synthesize and critique what is known about implementation strategies to promote infection prevention and control for nurses. A total of 61 articles met the inclusion criteria for the final review. The articles were evaluated using the Joanna Briggs Institute's (JBI) quality appraisal tools. Results were reported using PRISMA guidelines. Findings:<br><br>- Most studies were conducted in South Africa within the last 18 years and utilized a quasi-experimental design<br>- Few studies had sample populations comprising nurses only.<br>- The majority of studies focused on administrative precautions<br>- The most frequent implementation strategies reported were education, quality management, planning, and restructure<br>- Penetration and feasibility were the most common outcomes measured for both evidence-based practices (EBPs) and	Barrera-Cancedda AE, Riman KA, Shinnick JE, Buttenheim AM. Implementation strategies for infection prevention and control promotion for nurses in Sub-Saharan Africa: a systematic review. Implementation Sci. 2019;14:111.	Multiple

			implementation strategies used to implement the EBPs.		

Table S2. Intervention: Provide hands on patient safety training to healthcare workers

	Method	Description	Evidence Summary	Source	Location and Study Period
1	Short refresher training course	Safer Anaesthesia From Education (SAFE) is a refresher course to upgrade the skills of anesthesia providers in low-income countries.	A mixed-methods design based on the Kirkpatrick model for evaluating educational training courses was used to determine personal and organizational change at 4 months and 12-18 months, and evaluate knowledge and skill retention.<br>Findings:<br>- Knowledge and skills tests in areas related to obstetric anesthesia both showed an immediate improvement after the course that was sustained.<br>- Organizational culture change was seen at both 4 and 12–18 months, including improved teamwork, communication, and preparation.<br>- Resistance from peers, lack of senior support, and lack of resources were cited as barriers to change at 4 months, but at 12–18 months, very few interviewees mentioned lack of resources.	White MC, Rakotoarisoa T, Cox, NH, et al. A Mixed-Method Design Evaluation of the SAFE Obstetric Anaesthesia Course at 4 and 12–18 Months After Training in the Republic of Congo and Madagascar. Anesth Analg. 2019;*129*(6): 1707-1714.	Republic of Congo Madagascar<br><br>2014 -2016

2	Short training course	A four-day pilot training course on the World Health Organization Surgical Safety Checklist (SSC)	A mixed-methods design based on the Kirkpatrick model was used to evaluate responses to a self-reported questionnaire assessing six key process measures. Learning, behavior, organizational change and facilitators and inhibitors to change were evaluated with questionnaires, interviews and focus group discussion.<br>Findings:<br>- Over half the participants were following the six processes measures always or most of the time:<br>- confirmation of patient identity and the surgical procedure (57%)<br>- assessment of difficult intubation risk (72%)<br>- assessment of the risk of major blood loss (86%)<br>- antibiotic prophylaxis given before skin incision (86%)<br>- use of a pulse oximeter (86%)<br>- counting sponges and instruments (71%).<br>- All participants reported positive improvements in teamwork, organization and safe anesthesia.<br>- Most participants reported they worked in a helpful, supportive and respectful atmosphere; and could speak up if they saw something that might harm a patient.<br>- Less than half felt able to challenge those in authority.	White MC, Peterschmidt J, Callahan J, Fitzgerald JE, Close KL. Interval follow up of a 4-day pilot program to implement the WHO surgical safety checklist at a Congolese hospital. Global Health. 2017;13(1):42.	Republic of Congo<br><br>2014
3	In service training course	In-service infection prevention and patient safety training based on the 22 modules of the Ethiopian Ministry of Health's Infection Prevention and Patient Safety	In-service infection prevention and patient safety training was provided from March 21-28, 2019 at Goba Referral Hospital, Ethiopia. Pre-test and post-test scores using a paired t-test to estimate the mean difference.<br>Findings:<br>- Post test scores were higher (Mean = 6.16) than pre-test scores (Mean = 5.25)<br>- On average, healthcare workers who participated in the infection prevention and patient safety training and took the post-test demonstrated improved awareness of infection prevention	Sahiledengle B, Zenbaba D, Allison D. Enhancing Healthcare Workers' Infection Prevention and Patient Safety Awareness: In-service Training at Goba Referral Hospital. Ethiopian	Ethiopia<br><br>2019

		Training Resource Package		Health Care Quality Bulletin. 2019;1:95–100.	
4	Introduction of checklist based patient safety programme	Implementation of the Trauma Care Checklist (TCC) programme to improve adherence to defined patient care-related tasks during the initial resuscitation of severely injured patients.	Thirteen sites were recruited to participate in implementation of the WHO Trauma Care Checklist programme; 11 sites implemented the programme and collected data. Th effect was assessed through a pre-/post-intervention comparison using a stepped wedge design with randomly assigned start dates.<br>Findings:<br>- Improvement was found for 18 of 19 process measures, including (all $p < 0.05$):<br>- Post implementation there were greater odds of having abdominal examination (OR 3.26), chest auscultation (OR 2.68) and distal pulse examination (OR 2.33)	Lashoher A, Schneider EB, Juillard C, Stevens K, Colantuoni E, Berry WR, et al. Implementation of the World Health Organization Trauma Care Checklist Program in 11 Centers Across Multiple Economic Strata: Effect on Care Process Measures. World J Surg. 2017;41:954–962.	Cameroon, India, Pakistan, Rwanda, Vietnam<br><br>(Plus 4 upper-middle and high-income countries)<br><br>2010 - 2012

5	Simulation	A novel multiplayer screen-based simulation in a virtual world enabling the practice of team coordination with postpartum hemorrhage (PPH) in Uganda	The study explored whether a multiplayer screen-based simulation experience would increase learner confidence in the ability to manage PPH in Mulago, Uganda. Learners were trainees or professionals from obstetrics, midwifery, and anesthesiology. The study design was a simple fifteen question pre- and a post-intervention survey.<br>Findings:<br>- Combined confidence scores increased significantly overall following the simulation experience (pre = 7.83 ± 1.55, post = 8.95 ± 1.42, $p < 0.001$)<br>- Confidence scores increased individually in each of the three categories of Bloom's Taxonomy: affective, cognitive, and psychomotor.	Taekman JM, Foureman MF, Bulamba F, Steele M, Comstock E, Kintu A, et al. A Novel Multiplayer Screen-Based Simulation Experience for African Learners Improved Confidence in Management of Postpartum Hemorrhage. Front Public Health, 2017;5:248.	Uganda<br><br>2015
6	Simulation	A 3-hour simulation session for postgraduate surgical trainees in Rwanda using a high-fidelity, tissue-based model simulating the creation of an end ileostomy.	Participants were anonymously recorded completing the assigned task at three time points: prior to, immediately following, and 90 days following the simulation training.<br>Findings:<br>- Mean OSATS score improvement for participants who completed all the assessments was 6.1 points [95 % Confidence Interval (CI) 2.2–9.9, p = 0.005].<br>- Improvement was sustained over a 90-day period with a mean improvement of 4.1 points between the first and third attempts (95 % CI 0.3–7.9, p = 0.038).<br>- Simulation training was effective in both study sites, though most gains occurred with junior-level learners, with a mean improvement of 8.3 points (95 % CI 5.1–11.6, p < 0.001).<br>- Significant improvements were not identified for senior-level learners.	Tansley G, Bailey JG, Gu Y, Murray M, Livingston P, Georges N, et al. Efficacy of Surgical Simulation Training in a Low-Income Country. World J Surg. 2016;40:2643–2649.	Rwanda<br><br>(Plus Canada)<br><br>2014

7	Simulation	The Vital Anaesthesia Simulation Training (VAST) course is a 3-day immersive simulation-based programme concentrating on core clinical challenges and non-technical skills required by anaesthesia providers in low-resource settings.	A mixed-methods study evaluated the impact of VAST in Rwanda. Anaesthetists' Non-Technical Skills scores were quantitatively assessed for 30 course participants at three time points (pre-, post-, and 4 months after VAST). Qualitative data were gathered during focus groups (4 months after VAST) to learn of participants' experiences implementing new knowledge.<br>Findings:<br>- The ANTS total scores improved from pre- (11.0 [2.3]) (mean [standard deviation]) to post-test (14.0 [1.6]), and improvements were maintained at retention (14.2 [1.7]).<br>- The key theme that emerged during focus groups discussions was that the use of cognitive aids and clinical algorithms, repeated and reinforced across simulated scenarios, encouraged a systematic approach to patient care.<br>- Participants attributed the systematic approach to improving their problem-solving skills and confidence.<br>- Participants found value in well-functioning teams and shared decision-making.<br>- After VAST, participants described empowerment to advocate for better patient care and system improvement.	Mossenson A, Tuyishime E, Rawson D, Mukwesi C, Whynot S, Mackinnon SP, et al. Promoting anaesthesia providers' non-technical skills through the Vital Anaesthesia Simulation Training (VAST) course in a low-resource setting<br>Br J Anaesth. 2020;124(2): 206-213.	Rwanda<br><br>2018-2019

8	Short training programme (and ongoing 1-2-1 mentoring)	Short training programme in sterile processing (2 weeks) for staff in Benin hospitals which covered microbiology, cleaning and decontamination, sterilization and others	A mixed-methods study evaluated the impact of a short training programme. The SP training programme was undertaken in 3 hospitals serving 1.2m people. Before training all completed SP knowledge tests and repeated them 4 months after training. Semi structured interviews also carried out.<br>Findings:<br>5 themes identified:<br>- *We changed how things were are done*: now wore gloves, used 3-bucket system to clean instruments, changed cleaning habits<br>- *We changed our way of seeing things:* greater understanding of why and how SP should be done<br>- *Now we pay attention*: inspection became part of cleaning process<br>- *Decreased surgical site infections:* nine participants reported decrease in surgical site infections<br>- *Resource concerns:* lack of resources reported as barriers to implementing recommended practices, including lack of equipment and funds<br>- Knowledge test results: mean pre-course test score 57% and post course was 71% ($p<0.001$)	Fast O, Fast C, Fast D, Veltjens S, Salami Z, White M. Mixed methods evaluation of the impact of a short-term training program on sterile processing knowledge, practice and attitude in three hospitals in Benin. Antimicrob Resist Infect Control. 2018;7(20).	Benin<br><br>2016-2017

9	Smartphone App	Simulation training for tendon repair via smartphone app in Rwanda	Randomised control trial of surgical residents' utilisation of Touch Surgery (TS) smartphone application. Effect on cognitive and technical skills previously unknown in LMICs. Participants were surgery residents from University of Rwanda in general surgery, orthopaedics, urology and neurosurgery. Residents randomised to study tendon repair via textbook or the app. All necessary materials for the simulation were provided by the research team. As no validated rubrics for tendon repair, scoring rubric created for study.<br>Findings:<br>- Operative skills measured by tendon repair simulation overall rubric score was 22.43 (89.71%) for TS users and 15.85 (63.4%) for textbook users (p <0.001).<br>- Difference in cognitive skills measures by comparing the percentage change in pre multiple-choice and post multiple-choice questionnaire total scores was 15.9% (p=0.304) for the textbook group and 38.6% (p<0.001) for the TS group.	Juru Bunogerane G, Taylor K, Lin Y, Costas-Chavarri A. Using Touch Surgery to Improve Surgical Education in Low- and Middle-Income Settings: A Randomized Control Trial. J Surg Educ. 2018;75(1):231-237.	Rwanda<br><br>2017

Table S3. Intervention: Introduce a context-specific risk management tool

	Method	Description	Evidence Summary	Source	Location and Study Period

1	Maternal near-miss case review (NMCR)	The facility-based individual NMCR cycle is a type of criterion-based audit seeking to improve maternal and perinatal healthcare and outcomes by conducting a review, at hospital level, of the care provided to maternal near-miss cases.	A systematic review was conducted to collect evidence on the effectiveness of the NMCR on QoC and maternal and perinatal health outcomes in low-income and middle-income countries (LMICs). 17 studies from 11 countries were included. Findings:<br>- Maternal mortality measured before and after the implementation of the NMCR cycle significantly decreased (OR 0.77, 95% CI 0.61 to 0.98, eight studies, 55 573 043 women; I^2=39%).<br>- A statistically significant reduction in the incidence of uterine rupture, postpartum haemorrhage and maternal sepsis was observed in three out of six studies.<br>- Ten studies reporting on maternal care process all showed some significant improvement when measured against predefined standards.<br>- All studies reported that the NMCR resulted in some amelioration of the facility structure (physical structure, staffing, equipment, training, organisation of care).<br>- Newborn outcomes were overall poorly reported; four studies showed no significant difference in perinatal mortality.	Lazzerini M, Richardson S, Ciardelli V, Erenbourg A. Effectiveness of the facility-based maternal near-miss case reviews in improving maternal and newborn quality of care in low-income and middle-income countries: a systematic review. BMJ Open. 2018;*8*(4):e019787.	Ghana<br>Ethiopia<br>Malawi<br>Nigeria<br>Tanzania<br>Uganda<br>Vietnam<br><br>(Plus Kazakhstan, Moldova, Malaysia, Jamaica)<br><br>2001 - 2012
2	Paediatric Hospital Reporting (PHR) programme	The PHR programme was set up to report the diagnoses included in the WHO Integrated Management of Childhood Illness (IMCI), Hospital Care for Children guidelines, and the PNG Standard Treatment Manual for	The data were collected and summarised in individual hospitals, where outcome data were automatically calculated on a summary sheet for any given time period. The data was used at a local level to monitor activity and disease patterns, for auditing and to plan local interventions. Findings:<br>- The data informed the National Policy and Plan for Child Health,<br>- The data triggered the implementation of a process of clinical quality improvement and other interventions to reduce mortality in the neediest areas, focusing on diseases with the highest burdens.	Duke T, Yano E, Hutchinson A, Hwaihwanje I, Aipit J, Tovilu M et al. Large-scale data reporting of paediatric morbidity and mortality in developing countries: it can be done. Arch Dis Child. 2016;101:392-397.	Papua New Guinea<br><br>2009-2014

		Common Illnesses.			
3	Standardised child death review process	A standardised child death review process introduced in the paediatric department of the National Hospital in Honiara, Solomon Islands	The child death auditing process was assessed through systematic observations made at each of the weekly meetings using the following standards for evaluation: (1) adapted WHO tools for paediatric auditing; (2) the five stages of the audit cycle; (3) published principles of paediatric audit; and (4) WHO and Solomon Islands national clinical standards of Hospital Care for Children.<br>Findings:<br>- Areas of the process identified for improvement were use of a more systematic classification of causes of death, inclusion of social risk factors and community problems in the modifiable factors, and more follow-up with implementation of action plans<br>- Other areas for improvement were in communication, clinical assessment/treatment, availability of lab tests, antenatal clinic attendance and equipment for high dependency neonatal and paediatric care.<br>- Many of the changes recommended by audit require a quality improvement team to implement.	Sandakabatu M, Nasi T, Titiulu C, Duke T. Evaluating the process and outcomes of child death review in the Solomon Islands. Arch Dis Child. 2018;103(7); 685–690.	Solomon Islands<br><br>2017

4	Implementing best available evidence for practice	Implementation of the best available evidence for practice was used to enhance sharps injury prevention among healthcare workers in the Surgical Department of the Jimma University Specialized Hospital.	Audits were conducted using the Joanna Briggs Institute clinical audit tool Practical Application of Clinical Evidence system (JBI-PACES). Post intervention audit, which was a replication of the pre-audit, was carried out from 1 - 6 October 1 2012. The results were then used to undertake the Getting Research into Practice (GRIP) program.<br>Findings:<br>- A moderate improvement (29%) was observed on the compliance rate of criteria 1 (sharp containers/safety boxes not filled above the indicated line).<br>- A significant improvement (20% to 72%) was achieved in criteria 2 (positioning the sharps containers out of reach of children at a height safe for disposal).<br>- The finding from criteria 3 (sharps containers are positioned where they can be easily accessed) showed a 51% improvement.<br>- The lowest improvement (4% to 12%) was achieved in criteria 4 (incidence reporting by healthcare workers).<br>- A 100% compliance rate was achieved in criteria 5 (staff education).<br>- Moderate improvement was achieved in criteria 6 (used sharps are discarded into sharps containers at the point of use) with an 80% compliance rate.	Admassu B, Abdulahi M, Fogi T, Egziabher H. Sharps injury prevention among healthcare workers in the surgery department, Jimma University Specialized Hospital, Jimma: A best practice implementation project. JBI Fellows Monographs 2012. 2012. Available: http://joannabriggs-webdev.org/assets/docs/publications/FellowsReports/Fellows-Report_Africa_2012.pdf#page=97	Ethiopia<br><br>2012

5	Criteria based audit	A criteria based audit of the diagnosis and management of obstructed labour in Tanzania	The study evaluated the impact of a criteria-based audit of the diagnosis and management of obstructed labour in a low-resource setting (Tanzania). The implemented interventions included but were not limited to introducing standard guidelines for diagnosis and management of obstructed labour, agreeing on mandatory review by specialists for cases that are assigned caesarean section, retraining and supervision on use and interpretation of partograph and, strengthening teamwork between staff.<br>Findings:<br>- Implementing the new criteria improved the diagnosis from 74% to 81% ($p = 0.049$) and also the management of obstructed labour from 4.2% at baseline audit to 9.2% at re-audit ($p = 0.025$).<br>- Improved detection of prolonged labour through heightened observation of regular contractions, protracted cervical dilatation, protracted descent of presenting part, arrested cervical dilation, and severe moulding contributed to improved standards of diagnosis (all $p < 0.04$).<br>- Patient reviews by senior obstetricians increased from 34% to 43% ($p = 0.045$) and reduced time for c-section intervention from the median time of 120 to 90 minutes ($p = 0.001$) improved management (all $p < 0.05$).<br>- Perinatal outcomes, neonatal distress and fresh stillbirths, were reduced from 16% to. 8.8% ($p = 0.01$).	Mgaya AH, Kidanto HL, Nystrom L, Esse´n B. Improving Standards of Care in Obstructed Labour: A Criteria-Based Audit at a Referral Hospital in a Low-Resource Setting in Tanzania. PLoS ONE. 2016;11(11): e0166619.	Tanzania<br><br>2013-2014

6	The Alphabet Strategy	The Alphabet Strategy (AS) is a diabetes care checklist ensuring “important, simple things are done right all the time” implemented across 32 countries.	Audit data was collected from 52 centres across 32 countries. Data from 4537 patients were converted to Quality and Outcome Framework (QOF) scores to enable inter-centre comparison. A retrospective audit on 100 randomly selected case notes was conducted prior to AS implementation in a diabetes outpatient clinic in India, followed by a prospective audit after four months to assess its impact on care quality. Findings:<br>- There was significant improvement in the documentation of total cholesterol, serum lipid profile, renal function and proteinuria, glycaemia and uptake of guardian drugs ($p < 0.001$).<br>- Quality and Outcome Framework (QOF) scores increased by 36% from 45 to 61 ($p < 0.001$).	Lee JD, Saravanan P, Varadhan L, Morrissey JR, Patel V. Quality of diabetes care worldwide and feasibility of implementation of the Alphabet Strategy: GAIA project (Global Alphabet Strategy Implementation Audit). BMC Health Serv Res. 2014;14:467.	India<br><br>(Though the wider study was across LMIC countries)
7	Standardised auditing system	A standardised auditing system on the clinical auditing completion rate and accreditation standards compliance was created in Rwanda	A pre- and post-intervention study was used to examine the impact of creating a standardised auditing system on the clinical auditing completion rate and accreditation standards compliance. Two measures collected were:<br>1. Department clinical auditing completion rate<br>2. The COHSASA (Council for Health Service Accreditation of South Africa) standards compliance rate<br>Findings:<br>- Completion rate of monthly audit increased from 57% to 96% ($p<0.000$).<br>- Hospital wide average accreditation standards compliance rate also improved from 37% to 60% ($p=0.000$).<br>- Providing departments user-friendly clinical documentation auditing tool can enhance the completion rate of audit reporting at no additional cost to the hospital.	Kamanzi J, Mengentta A, Nsabiyumva W, Sendegeya A, Wong R. Improving Clinical Documentation through Monthly Audits in Butare Teaching Hospital, Rwanda. Journal of Service Science and Management. 2015;8:860-867.	Rwanda<br><br>2014

Table S4. Intervention: Implement priority patient safety interventions at the point of care

	Method	Description	Evidence Summary	Source	Location and Study Period
1	New medicine policy in public sector	Review of outcomes of new medicine policy implemented in Burkina Faso	Review of outcomes of new medicine policy implemented in 1995 on availability of injection services and estimation of proportion of prescriptions including at least one injection in 52 healthcare facilities evaluated.<br>Findings:<br>96% of these healthcare facilities equipped with a pharmaceutical depot setting syringes and needles, 74% of these had been established between 1995-2000. Of the 50 facilities, 96% had single use 5ml syringes available. Proportion of prescriptions including at least one injection remained stable between 1995-2000 (26.5%-23.8%).	Logez S, Hutin Y, Somda P. Safer injections following a new national medicine policy in the public sector, Burkina Faso 1995-2000. BMC Public Health. 2005;5:136.	Burkina Faso<br><br>2001
2	WHO Safe Childbirth checklist	Training for all clinical staff on WHO Safe Childbirth checklist and posters around the maternity unit.	Pre- and post-intervention study of implementation of WHO Safe Childbirth checklist in a district hospital. Implementation included providing training for all clinical staff and posters around the maternity unit.WHO Safe Childbirth Checklist (SCC) low cost tool designed to ensure birth attendants perform 29 essential birth practices (EBP) at four critical periods in the birth continuum. The study focused on observations of 29 EBPs done before and after implementation.<br>Findings:<br>- 391 birth events observed pre-intervention and 289 post-intervention.<br>- Overall EBP compliance rate increased from 46% pre-intervention to 56% post intervention (p=0.005) with significant improvement in 11 of EBPs.	Tuyishime E, Park P, Rouleau D. Implementing the World Health Organisation safe childbirth checklist in a district Hospital in Rwanda: a pre- and post-intervention study. Matern Health Neonatol Perinatol. 2018;4:7.	Rwanda<br><br>2017

3	Multiple surgical site infection (SSI) preventi on measure s	Multiple surgical site infection (SSI) prevention measures, combined with improvement of teamwork and safety climate in five African hospitals.	A before-after cohort study of multimodal intervention to improve incidence of surgical site infections. Four hospitals completed baseline and follow-up. Three provided suitable data. 4322 operations were followed up.<br>Findings:<br>- SSI cumulative incidence significantly decreased post intervention from 8% to 3.8% (p=<0.0001) which persisted in the sustainability period (3.9%)<br>- Substantial improvement in compliance with prevention measures was observed in follow- up and sustainability period<br>- The likelihood of SSI during follow up was lower (OR= 0.40, p<0.0001), but the likelihood of death was no significantly reduced (OR=0.72, p=0.2360)	Allegranzi B, Aiken AM, Kubilay NZ. A multimodal infection control and patient safety intervention to reduce surgical site infections in Africa: a multicentre, before-after, cohort study. Lancet Infect Dis. 2018;18:507-15.	Kenya, Uganda (two hospitals), Zambia, Zimbabwe<br><br>2013-2015
4	Surgical Unit-Based Safety Program (SUSP)	Multimodal strategy including multiple SSI prevention measures alongside adaptive approach to teamwork and leadership to enhance patient safety culture.	Qualitative study with individuals from hospitals participating in SUSP. Interviews with individuals from all hospitals participating in WHO-coordinated before-after SUSP study. Semi-structured interviews with 2-3 individuals from each of 5 hospitals.<br>Findings:<br>- Identified facilitators included: influential individuals, peer-to-peer learning, implementation fitness, timely feedback<br>- Participating hospitals were able to benefit from SUSP programme structure and adaptive tools to help change perceptions of patient safety.<br>- Acomplex combination of contextual factors, rather than isolated barriers or facilitators, influenced the success of implementation.	Clack L, Willi U, Berenholtz S. Implementati on of a surgical unit-based safety program in African hospitals: a multicentre qualitative study. Antimicrob Resist Infect Control. 2019;8:91.	Kenya, Uganda (two hospitals), Zambia and Zimbabwe<br><br>Initiated 2013

5	Medication safety training for nurses	A two-day nurse medication safety CPD session was developed and refined during 2015 and 2016 in Mozambique.	In phase one, partners developed a training package, which was delivered to 87 Portuguese-speaking nurses. In phase two, the refined training was delivered to 36 nurses in Mozambique and recoded by health psychologists. Measures of participant confidence and intentions to make changes to healthcare practice were collected, as well as qualitative data through post-training questions and 12 short follow-up participant interviews. Findings:<br>- Participants reported high confidence before and after the training (p = 0.25)<br>- Intentions to use calculators to check drug calculations significantly increased (p = 0.031).<br>- Qualitative data suggested the training was acceptable, enjoyable and led to practice changes, through improved capability, opportunity and motivation.<br>Opportunity barriers to medication safety were highlighted.	Bull ER, Mason C, Junior FD, Santos LV, Scott A, Ademokun D, et al. Developing nurse medication safety training in a health partnership in Mozambique using behavioural science. Global Health. 2017;13(1):45.	Mozambique<br><br>Initiated 2015
6	Electronic prescribing	Introduction of an electronic inpatient record and prescribing system in Pakistan	Prospective review of medication and discharge medication charts before and after the introduction of an electronic inpatient record and prescribing system. Inpatient records and discharge medication sheets were reviewed for prescribing errors before and after the installation of the electronic prescribing system in 11 wards. Findings:<br>- The overall medication error rates for inpatients were 22·6% and 8·2% throughout paper-based and electronic prescribing, respectively<br>- The overall medication error rates for discharge patients were 16·9% and 4·4% during paper-based and electronic prescribing, respectively.	Shawahna R, Rahman N-U, Ahmad M, Debray M, Yliperttula M, Declèves X. Electronic prescribing reduces prescribing error in public hospitals. J Clin Nurs. 2011;20:3233-3245.	Pakistan<br><br>2011
